# The up‐regulation of NDRG1 by HIF counteracts the cancer‐promoting effect of HIF in VHL‐deficient clear cell renal cell carcinoma

**DOI:** 10.1111/cpr.12853

**Published:** 2020-06-14

**Authors:** Zheng‐Yan Zhang, Shi‐Long Zhang, Hui‐Ling Chen, Yu‐Qin Mao, Zhan‐Ming Li, Chao‐Yue Kong, Bing Han, Jin Zhang, Yong‐Hui Chen, Wei Xue, Wei Zhai, Li‐Shun Wang

**Affiliations:** ^1^ Key Laboratory of Whole‐Period Monitoring and Precise Intervention of Digestive Cancer (SMHC) Minhang Hospital Fudan University Shanghai China; ^2^ Institute of Fudan‐Minhang Academic Health System Minhang Hospital Fudan University Shanghai China; ^3^ Department of Urology Renji Hospital Shanghai Jiao Tong University School of Medicine Shanghai China

**Keywords:** clear cell renal cell carcinoma, HIF‐1/2α, metastasis, NDRG1, proliferation

## Abstract

**Background:**

Hypoxia‐inducible factors (HIFs) are thought to play important roles in the carcinogenesis and progression of VHL‐deficient clear cell renal cell carcinoma (ccRCC).

**Methods:**

The roles of HIF‐1/2α in VHL‐deficient clear cell renal cell carcinoma were evaluated by bioinformatics analysis, immunohistochemistry staining and Kaplan‐Meier survival analysis. The downstream genes that counteract the cancer‐promoting effect of HIF were analysed by unbiased proteomics and verified by in vitro and in vivo assays.

**Results:**

There was no correlation between the high protein level of HIF‐1/2α and the poor prognosis of ccRCC patients in our large set of clinical data. Furthermore, NDRG1 was found to be up‐regulated by both HIF‐1α and −2α at the cellular level and in ccRCC tissues. Intriguingly, the high NDRG1 expression was correlated with lower Furman grade, TNM stage and longer survival for ccRCC patients compared with the low NDRG1 expression. In addition, NDRG1 suppressed the expression of series oncogenes as well as the proliferation, metastasis and invasion of VHL‐deficient ccRCC cells in vitro and vivo.

**Conclusions:**

Our study demonstrated that HIF downstream gene of NDRG1 may counteract the cancer‐promoting effect of HIF. These results provided evidence that NDRG1 may be a potential prognostic biomarker as well as a therapeutic target in ccRCC.

## 1 INTRODUCTION

Hypoxia‐inducible factor (HIF), which is a heterodimeric basic helix‐loop‐helix/PAS protein, consists of an oxygen‐sensitive alpha subunit (HIF‐1α, HIF‐2α or HIF‐3α) and a stable beta subunit (HIF‐1β, also known as aryl‐hydrocarbon receptor nuclear translocator (ARNT)). Under normoxic conditions, the HIF‐1/‐2α subunits are hydroxylated by prolyl hydroxylase enzymes (EGLN, also known as PHD) through oxygen.^1^ Once hydroxylated, HIF‐α is subjected to conjugate with the von Hippel‐Lindau tumour suppressor protein (pVHL), which is the substrate‐recognition component of E3 ubiquitin ligase complex. pVHL recruits an E3 ubiquitin ligase that catalyses polyubiquitination of HIF‐α, thereby targets it for proteasomal degradation.^2^, ^3^, ^4^

There are hypoxia and/or anoxia regions in 50% to 60% of solid tumours.^5^ Under hypoxic conditions, PHD activity is inhibited and the pVHL fails to recognize HIF‐α and make it ubiquitylation, resulting in the accumulation of HIF‐1/2α subunits in the cytoplasm.^3^ Then, HIF‐1/2α subunits translocate to the nucleus, form dimerization with HIF‐1β and subsequently activate directly or indirectly numerous genes that are involved in cell proliferation, migration and invasion, angiogenesis, metabolic shift towards glycolysis and survival. For example, the production of vascular endothelial growth factor (VEGF), PDGF‐β and transforming growth factor‐alpha (TGF‐α) and several glycolytic enzymes is up‐regulated by HIF, which endow cancer cells surviving advantages under hypoxia conditions.^6^

HIF‐1/2α have been associated with poor prognosis in a broad range of human cancers including astrocytoma, breast, melanoma, ovarian and prostate cancers.^7^ The critical role of HIF in cancer has led to its recent recognition as an ideal target for small molecule interventions. Many of the novel anti‐cancer drugs have been developed to inhibit HIF activity directly or indirectly. HDAC inhibitors and compounds have been shown to increase HIF‐α degradation. And DNA intercalating drugs, including echinomycin and anthracyclines, such as doxorubicin and daunorubicin inhibit HIF transcriptional activity by blocking its binding to DNA. Receptor tyrosine kinases inhibitors (such as gefitinib and erlotinib) and mTOR inhibitors (such as rapamycin) are thought to reduce tumour angiogenesis by indirectly reducing the synthesis of HIF‐α subunits.^8^, ^9^, ^10^, ^11^

Kidney cancer is one of the most common cancers. The incidence and mortality of kidney cancer have been increasing in many countries. Renal cell carcinoma (RCC) is the most prevalent subtype of kidney cancer (90%). About 75%‐80% of adult RCC are clear cell renal cell carcinomas (ccRCCs). Importantly, more than 90% of ccRCC tumours harbour biallelic inactivation of VHL via point mutation, deletion or methylation, which occur at the earliest stage of tumour formation.^12^ In ccRCC, the alterations of VHL mostly disrupt the function of pVHL so that HIF‐1/2ɑ cannot be degraded and are accumulated in the cancer cell even under normoxic conditions. Inactivation of VHL is thought to be an early event in the pathogenesis of ccRCC.^13^ But many molecular studies have supported the crucial role of HIF‐1/2α in the carcinogenesis and progression of ccRCC.^14^, ^15^, ^16^

Surprisingly, in this study we found that there was no correlation between the high protein level of HIF‐1/2α and the poor prognosis of ccRCC patients in our large set of clinical data. Furthermore, N‐myc downstream‐regulated gene 1 (NDRG1) was found to be up‐regulated by HIF‐1/2α in ccRCC tissues and its high expression was correlated with longer survival for ccRCC patients compared with the low NDRG1 expression. Our findings indicated that the up‐regulation of NDRG1 by HIF‐1/2α may counteract the cancer‐promoting effect of HIF‐1/2α in VHL‐deficient ccRCC.

## 2 MATERIALS AND METHODS

### 2.1 Clinical samples

A total of 645 paraffin‐embedded tumour samples and 260 paired adjacent normal tissues were obtained from ccRCC patients who underwent partial or radical nephrectomy in Department of Urology, Shanghai Renji Hospital, Shanghai Jiaotong University (Shanghai, China).

### 2.2 Immunohistochemistry (IHC) analysis

For the immunohistochemistry procedure, formalin‐fixed paraffin‐embedded (FFPE) tissue sections were dewaxed and repaired using ethylenediaminetetraacetic acid antigen retrieval buffers. After pre‐treatment, tissues were incubated with anti‐NDRG1 antibody (Sigma‐Aldrich, HPA006881), HIF‐1α antibody (Novus, NB100‐105) or HIF‐2α antibody (Novus, NB100‐122) and then incubated with secondary antibody. Considering staining intensity and area extent, we adopted the German semi‐quantitative scoring system to evaluate the protein level. Every sample was given a score according to intensity of nuclear or cytoplasmic staining (no staining = 0; weak staining = 1; moderate staining = 2; strong staining = 3) and percentage of tumour cells with positive staining (0% = 0; 1%‐10% = 1; 11%‐50% = 2; 51%‐80% = 3; 81%‐100% = 4). We determined the final score by multiplying the intensity and extent of positivity scores of stained cells.

### 2.3 Cell cultures

786‐O and Caki‐1 RCC cell lines were bought from Cell Bank in the Chinese Academy of Sciences (Shanghai, China). 786‐O cells were cultured in RPMI‐1640 medium (Gibco, USA) with 10% foetal bovine serum (FBS, Gibco, USA) with 1% penicillin/streptomycin (P/S, Hyclone, USA). Caki‐1 cells were cultured in McCoy’5A (Invitrogen) with 10% FBS. RCC4/EV and RCC4/VHL cells were given by Dr JK Cheng in SJTU‐SM, that were cultured in Dulbecco's modified Eagle's medium (DMEM, Hyclone, USA) with 10% FBS with 1% P/S. All cell lines were cultured in 5% CO_2_ air at 37°C. There were no signs of mycoplasma contamination for all cell lines.

### 2.4 Western Blot (WB) analysis and antibodies

Proteins were extracted from cells by using RIPA buffer with protease inhibitors. A 20 µg of proteins was loaded and separated by electrophoresis. Then, proteins were transferred to nitrocellulose membrane. Next, 5% non‐fat milk in Tris‐buffered saline was used to block the membrane and immune‐blotted with a primary antibody at 4°C overnight and, finally, incubated with a secondary antibody for 1 hour at room temperature. Detection was performed by Super Signal West Pico Chemiluminescent Substrate Kit (Pierce, Rockford, IL). The expression of β‐actin was used as loading control. NDRG1 antibody (HPA006881) was from Sigma‐Aldrich. Anti‐HIF1α (NB100‐105) antibodies were from Novus Biologicals. VHL (68547S) and HIF2α (7096S) antibodies were from Cell Signaling Technology. HRP‐conjugated Affinipure goat anti‐mouse (SA00001‐1), anti‐rabbit (SA00001‐2) antibodies and β‐actin antibody (20536‐1‐AP) were purchased from Proteintech.

### 2.5 Quantitative real‐time PCR (qRT‐PCR)

Total RNA was extracted from cells using TRIzol® reagent (Life Technologies) following the manufacturer's instruction. Then, RNA was converted to complementary DNA (cDNA). Quantitative real‐time polymerase chain reaction (qRT‐PCR) was performed on cDNA using gene‐specific primers in the presence of SYBR Green (Applied BioSystems). Data S9 lists the gene‐specific primers used in this study. Each measurement was performed in triplicate with at least three independent experiments. Transcript levels were normalized with GAPDH, and relative mRNA levels in experimental samples were normalized to controls.

### 2.6 Lentiviral shRNA, siRNAs and transfection

Pairs of complementary oligonucleotides against VHL, HIF‐2α and NDRG1 were synthesized, annealed and ligated into pSIREN‐RetroQ (Clontech, Mountain View, CA, USA). The target sequence for VHL, HIF‐2α and NDRG1 was described on our previous research.^17^, ^18^ Expression lentivirus for NDRG1 and corresponding control empty vector was transfected in Caki‐1 cell lines according to the manufacturer's instructions. After transfection, the virus supernatant was collected, filter‐sterilized and added to cells in six‐well plate containing polybrane with a final concentration of 4 mg/mL. The stably transfected cells were selected by adding puromycin (2 mg/mL). The siRNA was either designed against the HIF‐1α gene (siHIF‐1α or siHIF‐1α NC). RCC4 cells were treated with siHIF‐1α or siRNA‐NC complexed with in vivo liposome at a final concentration of 100 nmol/L. After 24 hours, media was replaced with DMEM media with 10% serum.

### 2.7 Cell proliferation assays

Cells were plated in triplicate in 96‐well plates in 100 μL appropriate growth medium. At indicated time points, each well was pulsed by addition of 10 μL of CCK‐8 assay (Dojindo, Kumamoto, Japan), followed by incubation at 37°C for 3 hours. Absorbance readings at a wavelength of 450 nm were taken.

### 2.8 Colony formation assay

Cells (100 cells per well) were seeded in a six‐well plate and cultured for 10 days. Colonies were then fixed with 4% paraformaldehyde for 15 minutes and stained for 10 minutes with 0.5% crystal violet.

Wells were washed with water to remove excess dye; dried plates were taken images, and the number of colonies was counted.

### 2.9 Transwell assays

Invasion assays were performed using the BD BioCoat Matrigel Invasion Chamber (354 480, BD Bioscience) according to the manufacturer's instructions. The 8.0‐µm‐pore Transwell chambers (353 097, Corning) were used for cell migration assays. 1 × 10^5^ cells were implanted in triplicate into the upper chamber in 200 µL of serum‐free medium, respectively. Complete medium with 10% FBS was added to the bottom chamber as a chemical attractant to stimulate migration or invasion. Next incubate in an incubator at 37°C in 5% CO_2_. After 20 hours, cells on the lower surface were fixed, stained with 0.3% crystal violet and counted.

### 2.10 In vivo assay

Animal care and experiments were carried out in strict accordance with the “Guide for the Care and Use of Laboratory Animals” and the “Principles for the Utilization and Care of Vertebrate Animals” and were approved by the ethics committee of Fudan University. Four‐ to 6‐week‐old male nude mice were obtained from Shanghai Research Center for Model Organisms. A total of 1 × 10^6^ 786‐O/NC and 786‐O/shNDRG1‐2 cells were injected subcutaneously. Tumour growth was blinded to measure every day after 5 days. The volume of the tumour was calculated using the equation length × width × width/2.

Four‐week‐old male nude mice were provided by Beijing Vital River Laboratory Animal Technology Co. Ltd. (Beijing, China). 786‐O/NC, 786‐O/shNDRG1‐2/3 cells transfected with luciferase were injected into nude mice via tail vein (1 × 10^5^ cells per mouse). Tumours were imaged to observe luciferase expression on day 28 after tumour cell injection. Briefly, the mice were anesthetized and then injected (i.p.) with luciferin in a volume of 100 μL at a dose of 150 mg/kg. Images were captured using an IVIS‐Lumina II imaging system (Caliper, USA) at a peak time of 10‐15 minutes after injection.

### 2.11 Data analysis

Differentially expressed proteins and mRNA were determined based on fold change log2 (log2FC)> |0.5| and unpaired *t* test *P*‐value < .05 by using Scaffold 4 software (version4.7.2, Proteome Software Inc, Portland, OR, USA).^18^ Differentially methylated regions (DMRs) identification, the mapped reads were used for detection of DMRs with statistically significant. And differentially methylated genes were determined based on log2FC > 1 and *P*‐value < .05. The networks functional analyses and functional annotation and clustering were performed by using QIAGEN’s Ingenuity Pathway Analysis (IPA, 2019‐Summer, QIAGEN).

### 2.12 Statistical analysis

The pre‐treated level RNA‐seq data and corresponding clinical information of ccRCC patients were collected from The Cancer Genome Atlas (TCGA) database (http://cancergenome.nih.gov/). All statistical ANALYSIS was performed by using IBM SPSS Statistics (version 24.0) and GraphPad Prism (version 6.0).

## 3 RESULT

### 3.1 HIF‐1/2ɑ cannot indicate the ccRCC patient's prognosis

**FIGURE 1:**
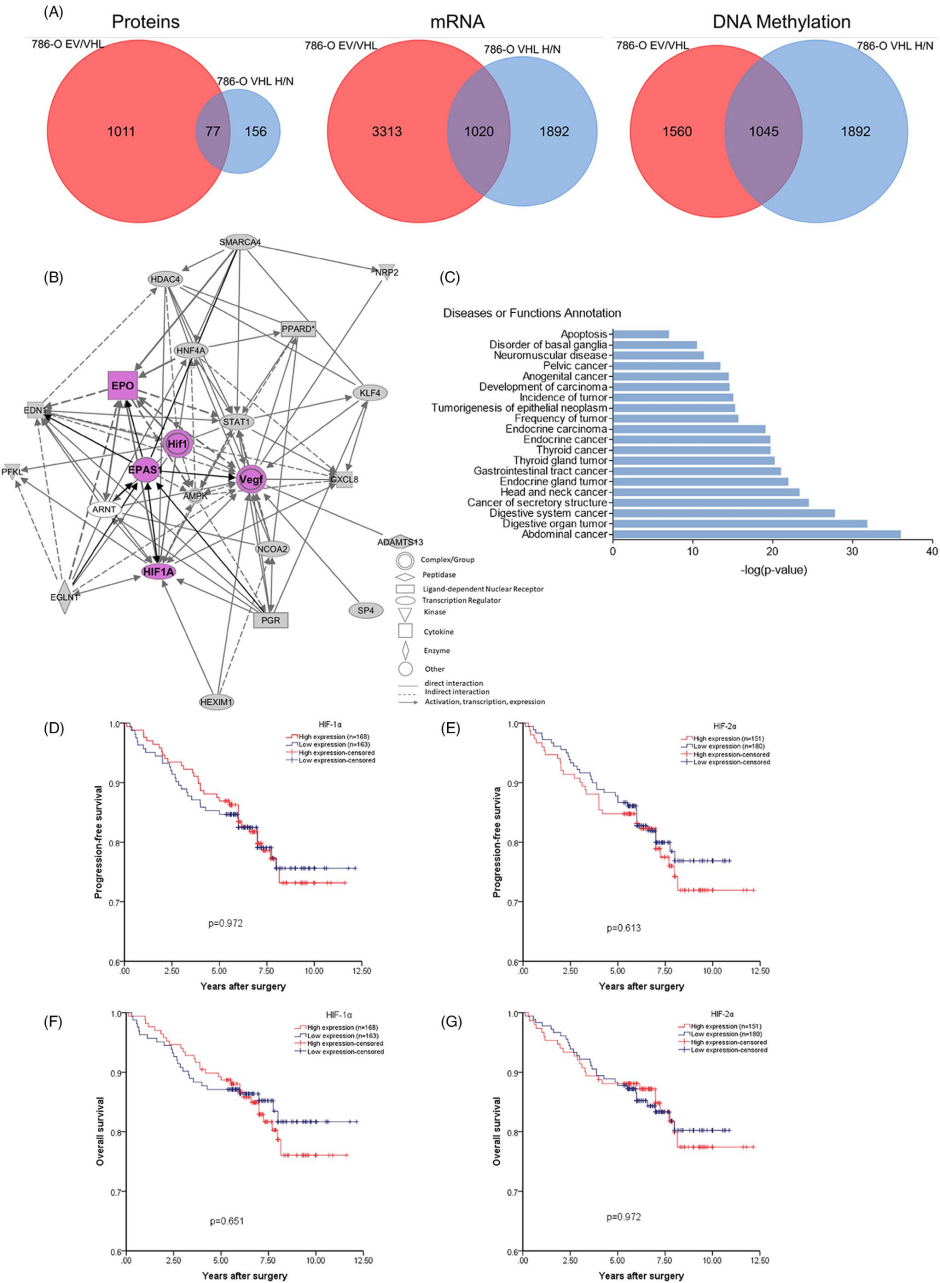
Neither HIF‐1α protein nor HIF‐2α protein can predict the ccRCC patient's prognosis. (A) The overlap from Venn diagram showing the proteins, mRNAs and methylated genes regulated by both VHL and hypoxia in 786‐O cells. EV, empty vector; H, hypoxia; N, normoxia. (B) The interacting network of HIF with the genes regulated by both VHL and hypoxia. (C) Diseases or functional clustering for genes regulated by both VHL and hypoxia. (D) Progression‐free survival and (F) overall survival among ccRCC patients with low or high protein levels of HIF‐1α by Kaplan‐Meier analysis. (E) Progression‐free survival and (G) overall survival among ccRCC patients with low or high protein levels of HIF‐2α by Kaplan‐Meier analysis. The log rank p‐value is reported

Given the critical role of HIF pathway in the carcinogenesis, we sought to evaluate the effect of the HIFs on the ccRCC patients systematically. To this aim, VHL was reintroduced into the VHL‐deficient 786‐O cell line, and its profiling of the protein, mRNA and DNA methylation were compared with VHL‐deficient 786‐O cell (Data S1). Similarly, the profiling of protein, mRNA and DNA methylation in 786‐O/VHL cells under hypoxic conditions was compared with 786‐O/VHL cells under normoxic conditions (Data S2), because VHL deficiency and hypoxia both fail to induce HIF‐1/2α proteasome degradation, which led to its accumulation in the cytoplasm. So, the common genes that were regulated by both VHL and hypoxia may be potential HIF‐regulated genes. From the overlap of Venn diagram in Figure 1A, there were 77 proteins regulated by both VHL and hypoxia in 786‐O cells (Data S3). And there were 1020 and 1045 genes, respectively, at mRNA and DNA methylation levels regulated by both VHL and hypoxia in 786‐O cells (Data S4 and S5). Next, we used QIAGEN’s Ingenuity Pathway bioinformatic analysis to analyse the interaction network of HIF‐1/2α with these potential HIF‐regulated genes. As shown in Figure 1B, we found that both HIF‐1α and HIF‐2α (EPAS1) have the ability to activate oncogenes deeply involved in cancer biology, such as VEGF, CXCL8 and PGR. And the bar chart of the functional annotation and clustering revealed that these potential HIF‐regulated genes were involved in series of cancers, such as gastrointestinal cancer, endocrine gland cancer, head and neck cancer (Figure 1C). The analysis of expression of putative HIF‐1/2α‐regulated gene in the 786‐O cells suggests that HIF‐1/2α may be associated with poorer overall survival in ccRCC.

Then, 331 specimens collected from ccRCC patients were stained with HIF‐1α/2α antibody, respectively, and scored according to the IHC as described in experimental procedures. Next, we used Kaplan‐Meier analysis to evaluate the relationship between HIF‐1/2α protein level and patient survival time. We found that high‐HIF‐1α protein level could not indicate good or poor survival compared with low‐HIF‐1ɑ protein level (progression‐free survival (PFS), *P* = .972; overall survival (OS), *P* = .651) (Figure 1D and F). Similarly, there was no statistical significance between HIF‐2α protein level and patient survival (PFS, *P* = .613; OS, *P* = .972) (Figure 1E and G). Further analysis revealed that there were no major statistical differences in baseline clinical pathologic parameters between the low and the high protein levels of HIF‐1/2α, except that HIF‐2α protein level is associated with Fuhrman grade (*P* = .012) (Tables 1 and 2). In conclusion, neither high protein level of HIF‐1α nor HIF‐2α can indicate the ccRCC patient's poor prognosis. However, from the bioinformatics results of the interaction network and bar chart in Figure 1B and C, the role of HIF in the development of ccRCC should be very important. So, we hypothesized that HIF activates downstream tumour suppressor genes in addition to the activating of oncogenic genes.

**TABLE 1:** Clinicopathological features of the patients and correlation with HIF‐1α protein

	Low expression	High expression	*P*‐value	Total
Age‐year				
Median	58	59	.531^a^	58
Range	24‐82	28‐82		24‐82
Age‐year‐no. (%)				
≤60	99 (61)	93 (55)	.322^b^	192 (58)
>60	64 (39)	75 (45)		139 (42)
Gender‐no. (%)				
Male	113 (69)	116 (69)	.956^b^	229 (69)
Female	50 (31)	52 (31)		102 (31)
Fuhrman Grade‐no. (%)				
I + II	94 (58)	112 (67)	.091^b^	206 (62)
III + IV	69 (42)	56 (33)		125 (38)
TNM stage‐no. (%)				
I + II	129 (79)	126 (75)	.37^b^	255 (77)
III + IV	34 (21)	42 (25)		76 (23)
pT stage‐no. (%)				
T1 + 2	134 (82)	135 (80)	.666^b^	269 (81)
T3 + 4	29 (18)	33 (20)		62 (19)
pN stage‐no. (%)				
N0	153 (94)	162 (96)	.277^b^	315 (95)
N1	10 (6)	6 (4)		16 (5)
pM stage‐no. (%)				
M0	129 (85)	146 (87)	.668^b^	285 (86)
M1	24 (15)	22 (13)		46 (14)
Tumour burden‐cm				
Median	4.2	4	.616^a^	4
Range	0.8‐12	1‐11.3		0.8‐12
Tumour burden‐cm‐no. (%)				
≤4	80 (49)	92 (55)	.301^b^	172 (52)
>4	83 (51)	76 (45)		159 (48)

^a^ Mann‐Whitney U test.

^b^ chi‐square test; TNM stage: AJCC renal cancer in 2010.

**TABLE 2:** Clinicopathological features of the patients and correlation with HIF‐2α protein

	Low expression	High expression	*P*‐value	Total
Age‐year				
Median	58	59	.679^a^	58
Range	24‐82	27‐82		24‐82
Age‐year‐no. (%)				
≤60	106 (59)	86 (57)	.722^b^	192 (58)
>60	74 (41)	65 (43)		139 (42)
Gender‐no. (%)				
Male	127 (71)	102 (68)	.555^b^	229 (69)
Female	53 (29)	49 (32)		102 (31)
Fuhrman Grade‐no. (%)				
I + II	123 (68)	83 (55)	.012^b^	206 (62)
III + IV	57 (32)	68 (45)		125 (38)
TNM stage‐no. (%)				
I + II	143 (79)	112 (74)	.256^b^	255 (77)
III + IV	37 (21)	39 (26)		76 (23)
pT stage‐no. (%)				
T1 + 2	148 (82)	121 (80)	.627^b^	269 (81)
T3 + 4	32 (18)	30 (20)		62 (19)
pN stage‐no. (%)				
N0	174 (97)	141 (93)	.165^b^	315 (95)
N1	6 (3)	10 (7)		16 (5)
pM stage‐no. (%)				
M0	156 (87)	129 (85)	.746^b^	285 (86)
M1	24 (13)	22 (15)		46 (14)
Tumour burden‐cm				
Median	4	4.5	.371^a^	4
Range	0.8‐11.3	0.8‐12		0.8‐12
Tumour burden‐cm‐no. (%)				
≤4	101 (56)	71 (47)	.099^b^	172 (52)
>4	79 (44)	80 (53)		159 (48)

^a^ Mann‐Whitney U test.

^b^ chi‐square test; TNM stage: AJCC renal cancer in 2010.

### 3.2 The gene‐regulatory network revealed that NDRG1 played a role in ccRCC

**FIGURE 2:**
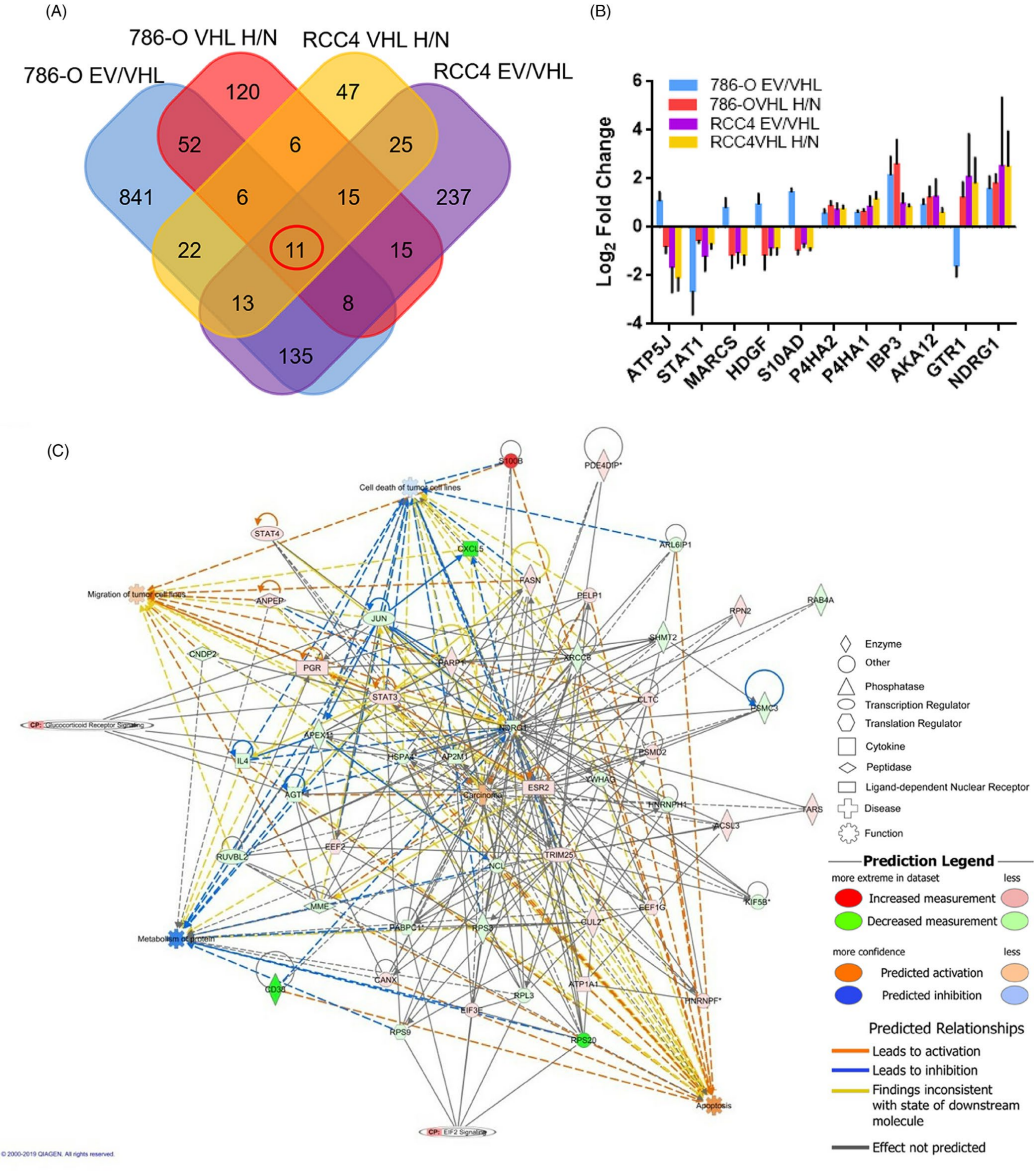
The gene‐regulatory network revealed that NDRG1 played a role in ccRCC. (A) Venn showed an overlap between the hypoxia‐related genes and genes regulated by VHL in the 786‐O and RCC4 cells. (B) The bar graph showed VHL and hypoxia‐regulated proteins in both RCC4 and 786‐O cells. EV, empty vector; H, hypoxia; N, normoxia. (C) The interaction map for NDRG1 and VHL‐regulated proteins, mRNA and methylation of genes indicated the important role of NDRG1 in ccRCC. Red nodes, genes were up‐regulated; green nodes, genes were down‐regulated. Colour depth indicated the changes of gene expression. The shape indicated the molecular class. The lines connecting the arrows indicated relationships between the molecules. Solid lines, direct interactions; dashed lines, indirect interactions

We then identified the potential tumour suppressor genes regulated by HIF‐1/2α in VHL‐deficient ccRCC cancer cells by subtractive proteomics strategy. In order to narrow down the range of genes regulated by HIF1/2α, we tried to identify the common gene in RCC4 and 786‐O cell lines. In addition to the 77 proteins regulated by VHL and hypoxia in 786‐O cells (Figure 2A and Data S1 and S2), we further identified the 64 proteins that were regulated by VHL and hypoxia in RCC4 cells through the direct comparisons between cells with EV and VHL transfection in normoxia, as well as cells with VHL transfection under normoxia and hypoxia conditions (Figure 2A and Data S6 and S7). Totally, there were 11 common proteins identified in these two cell lines through the overlap of Venn diagram (Figure 2A and Data S8), of which NDRG1 is the most significantly regulated protein in both RCC4 and 786‐O cells (Figure 2B). We hypothesized that NDRG1 might contribute to the inhibitory effect of HIF in tumour progression.

To determine whether NDRG1 suppresses tumour progression in ccRCC, we analysed the interaction of NDRG1 with the genes downstream of VHL. Three different levels of VHL‐regulated gene, including protein, mRNA and methylation of genes, were combined (Data S1). The interaction network between these VHL‐regulated genes and NDRG1 was shown in Figure 2C. NDRG1 was demonstrated to interact with series oncogenes, including JUN, STAT3, CXCL5, XRCC6, CD38, PGR and IL4. In addition, NDRG1 was shown to be down‐regulated by VHL and involved in carcinoma, apoptosis, cell death and migration of tumour cell lines. This suggests that NDRG1 played an important role in ccRCC.

### 3.3 NDRG1 was regulated by HIF‐1/2α

**FIGURE 3:**
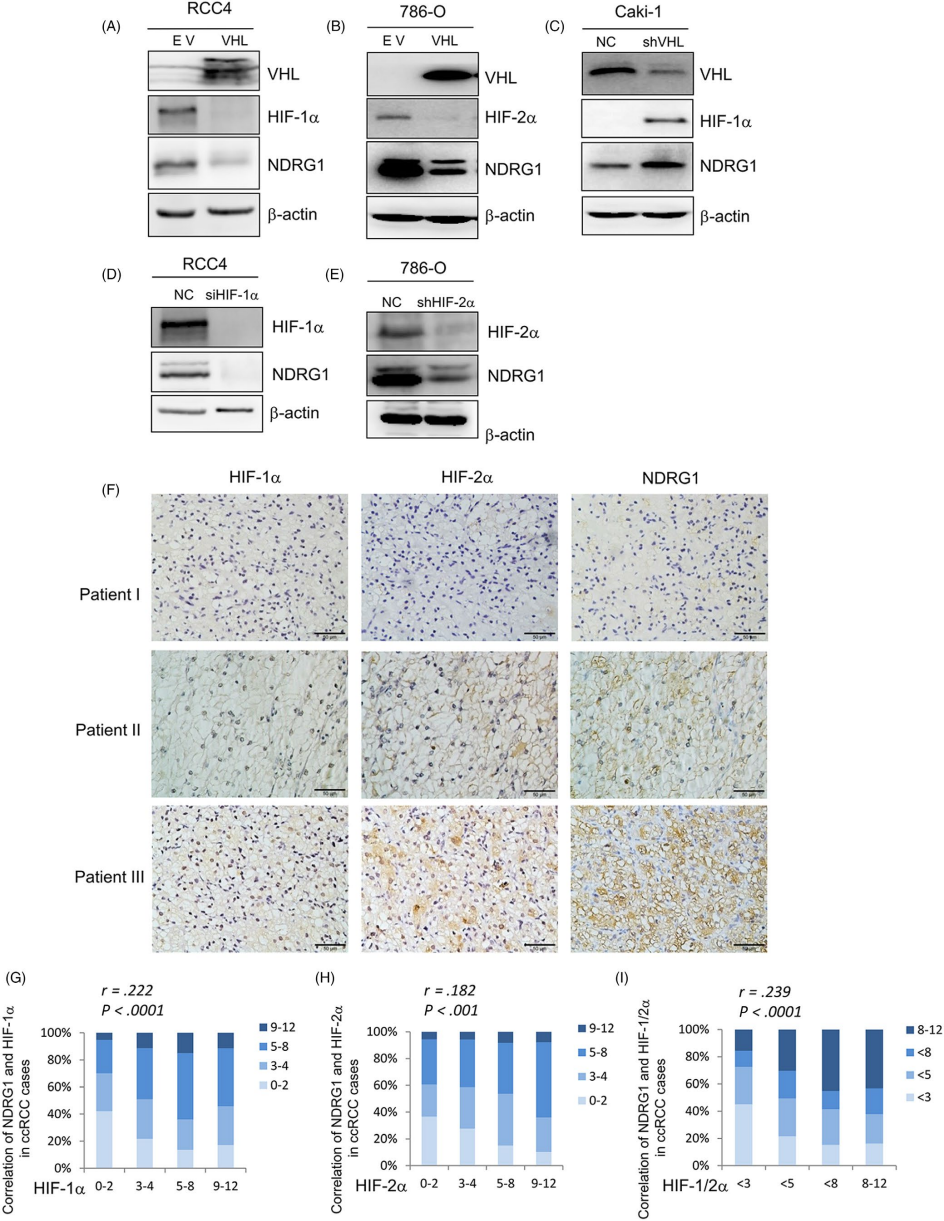
NDRG1 was regulated by HIF‐1/2α. (A‐E) The protein expression of the shown genes was detected by Western blot, respectively; β‐actin was used as the loading control. (A) RCC4 and 786‐O cells (B) were infected with VHL vector (VHL) or empty vector (EV). (C) Caki‐1 cells were transfected with VHL shRNAs (shVHL) or non‐specific control (NC). (D) RCC4 cells were infected with HIF‐1α siRNAs (siHIF‐1α) or non‐specific control (NC). (E) 786‐O cells were transfected with HIF‐2α shRNAs (shHIF‐2α) or non‐specific control (NC). (F) Representative images of HIF‐1α, HIF‐2α and NDRG1 protein expression from the IHC staining of human clear cell renal cell cancer specimens. Patient I: The protein levels of HIF‐1α, HIF‐2α and NDRG1 were low. Patient II: The protein levels of HIF‐1α, HIF‐2α and NDRG1 were medium. Patient III: The protein levels of HIF‐1α, HIF‐2α and NDRG1 were high (scale bar, 50 μm). (G and H) The protein level of NDRG1 is significantly correlated with HIF‐1α (G) and HIF‐2α (H). The protein levels were evaluated according to IHC scores, representing very low (score 0‐2), low (score 3‐4), high (score 5‐8) and strong (score 9‐12). The subjects were divided into four groups according to the IHC scores of HIFs in the tumours. (I) The protein level of NDRG1 is significantly correlated with the average of HIF‐1α and HIF‐2α in ccRCC tissues. The protein levels are evaluated according to IHC scores, representing very low (score < 3), low (score < 5), high (score < 8) and strong (score 8‐12). The subjects were divided into four groups according to the IHC scores of the average of HIF‐1α and HIF‐2α. Significance *p*‐values and *r* values according to Spearman's rank correlation

To validate the proteomic results that HIF‐1α/2α regulated NDRG1, reintroduction of VHL into VHL‐deficient 786‐O and RCC4 cells decreased the protein level of HIF‐1/2α. And we also found the protein levels of NDRG1 were also decreased (Figure 3A and B). Vice versa, both the protein of HIF‐1α and NDRG1 were significantly up‐regulated by silencing VHL expression in Caki‐1 cells (Figure 3C). Next, we disturbed the HIF‐1α expression in RCC4 cells and knocked down HIF‐2α in 786‐O cells. As shown in Figure 3D, the deletion of HIF‐1α in RCC4 decreased the expression of NDRG1 in protein levels. This is also true that the protein of NDRG1 was significantly down‐regulated by silencing HIF‐2α expression in 786‐O cells (Figure 3E).

To further validate whether the phenomenon could exist in clinic, we collected tumour tissue specimens from 331 patients of ccRCC. Three consecutive tumour sections were obtained from each tumour tissue specimen and were stained by anti‐NDRG1, anti‐HIF‐1α and 2α antibody, respectively, by IHC. As shown in Figure 3F, the low expression of NDRG1 was accompanied by low protein levels of HIF‐1α and 2α in IHC images of tumour tissues from Patient I. Moderate and high protein levels of HIF‐1α and 2α correspond to moderate and high NDRG1 expression, respectively, from Patients II and III. According to the scores of IHC, we quantified the protein levels of NDRG1, HIF‐1α and 2α to analyse the correlation between the expression of NDRG1 and HIF‐1α and 2α. The protein level of HIF‐1α was significantly correlated with NDRG1 expression (*r* = 0.222, *P* < .0001) (Figure 3G). Likewise, there was a positive correlation between the protein level of HIF‐2α and NDRG1 expression (*r* = 0.182, *P* < .001) (Figure 3H). Considering that both HIF‐1α and HIF‐2α have regulatory effect on NDRG1, next we used the average protein level of HIF‐1α and 2α to represent the level of HIFs. Similar to the above results, the average protein level of HIF‐1α and 2α was correlated with the expression of NDRG1 (*r* = 0.239, *P* < .0001) (Figure 3I). These results suggest that HIF‐1α and 2α regulate the expression of NDRG1 in ccRCC cells.

### 3.4 High NDRG1 predicted a better prognosis in ccRCC

**FIGURE 4:**
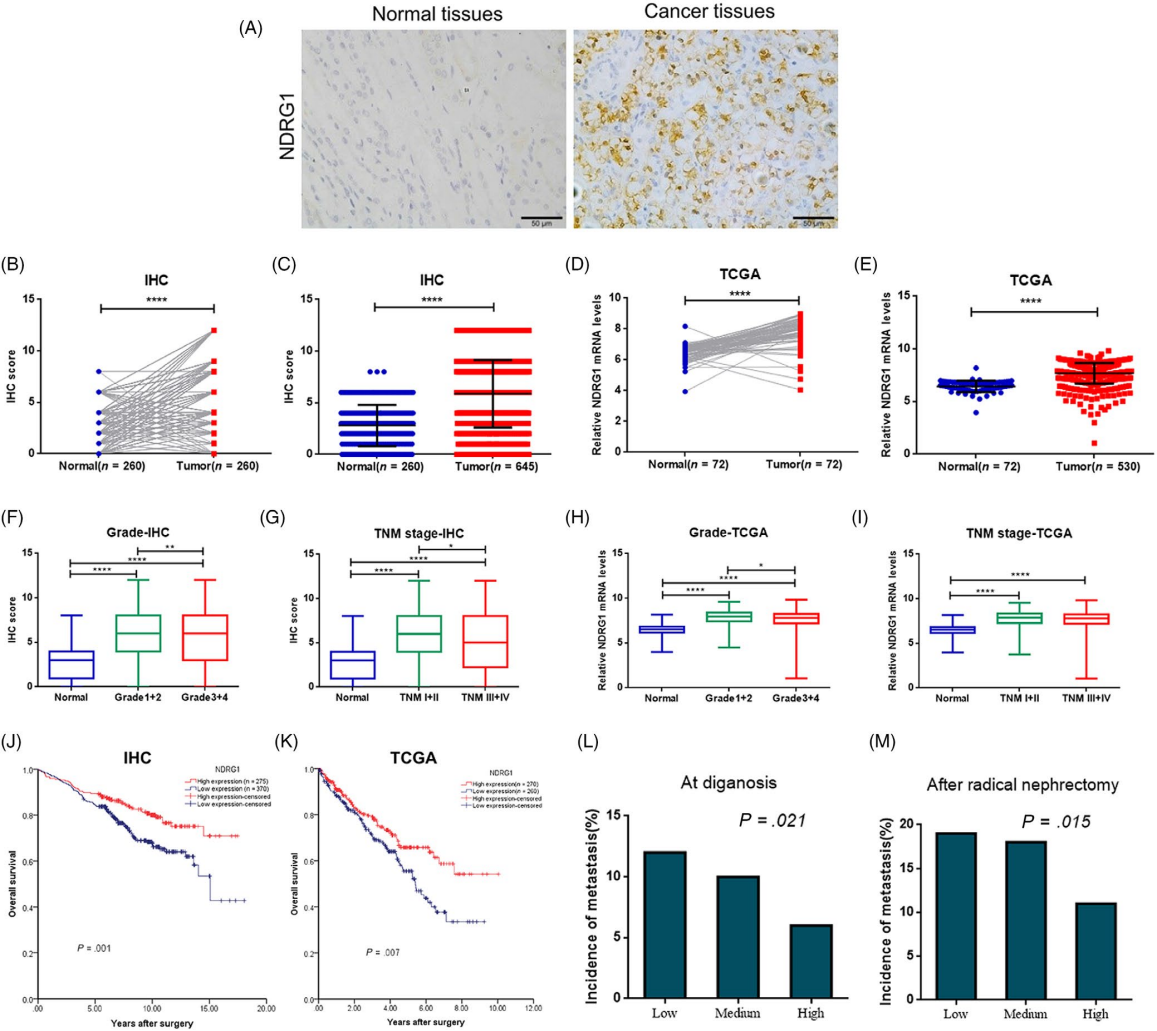
High NDRG1 predicted a better prognosis in ccRCC. (A) Representative images of NDRG1 expression from ccRCC tumour tissues and adjacent normal tissues by IH (scale bar, 50 μm). (B, C, F and G) The protein level of NDRG1 in normal and tumour tissues was quantified according to the score of IHC from our tissue microarray (TMA) data. (D and E, H and I) Relative mRNA levels of NDRG1 expression in normal and tumour tissues from TCGA data. (B and D) The NDRG1 expression in tumour tissue compared with corresponding adjacent normal tissue is shown. Significance according to Wilcoxon matched‐pairs signed rank test. (C and E) A Mann‐Whitney test was used to analyze differences of NDRG1 expression between in normal and tumour tissues. (F and H) Box plots comparing levels of NDRG1 expression in normal renal tissues, Grade1 + 2 and Grade3 + 4 carcinoma tissues by Mann‐Whitney test. (G and I) Box plots comparing levels of NDRG1 expression in normal renal tissues, TNM1 + 2 and TNM3 + 4 carcinoma tissues by Mann‐Whitney test. (J and K) Kaplan‐Meier curves were generated for patients with low and high levels of NDRG1 expression in TMA data (J) and TCGA data set (K), and with overall survival as the end point. The log rank p‐value is reported. (L and M) Frequency of patients with metastasis according to low, medium and high protein levels of NDRG1 as assessed by IHC score and analysed by linear‐by‐linear association. Low (score 0‐3), medium (score 4‐6), high (score 8‐12). (L) The incidence of metastasis at diagnosis (all patients: n = 645; low: n = 167; medium: n = 203; high: n = 275). (M) When excluding patients with metastasis at diagnosis, the incidence of metastasis after radical surgery (all patients: n = 589; low: n = 147; medium: n = 183; high: n = 259). *P < .05, **P < .01, ***P < .001, ****P < .0001 for Mann‐Whitney test

To determine the clinical implication of NDRG1 in ccRCC, we analysed the expression of NDRG1 in 260 ccRCC tissue samples and matched adjacent renal tissues by IHC according to Wilcoxon matched‐pairs signed rank test. We found that NDRG1 was predominantly localized in the cytoplasm (Figure 4A), and NDRG1 expression was increased in cancer issues compared with the paired adjacent tissue samples (*P* < .0001) (Figure 4B). Then, we analysed an additional cohort of ccRCC specimens. Higher expression of NDRG1 protein in cancer issues was further confirmed through Mann‐Whitney test (normal: n = 260, tumour: n = 645) (*P* < .0001) (Figure 4C). Consistently, mRNA levels of NDRG1 in 72 ccRCC patients were significantly higher compared to their paired normal counterparts in TCGA data set (*P* < .0001) (Figure 4D). And the mRNA level of NDRG1 from tumour tissues was higher than that from normal renal tissues (normal: n = 72, tumour: n = 530) (*P* < .0001) (Figure 4E). These data indicated that the expression of NDRG1 was increased in ccRCC.

Next, we examined whether NDRG1 expression was associated with clinicopathologic parameters. The protein expression of NDRG1 was significantly decreased in the higher ccRCC Fuhrman grade (*P* = .006) and TNM stage (*P* = .026) (Figure 4F and G, Table 3). In detail, the NDRG1 protein expression was negatively correlated with cancer pT stage (*P* = .001), pM stage (*P* = .026) and tumour size (*P* = .026) (Table 3). We also observed that the mRNA level of NDRG1 expression was significantly lower in higher Fuhrman grade (*P* = .038, Figure 4H and Table 4) except TNM stage (Figure 4I, Table 4). Furthermore, we evaluated the relationship between NDRG1 expression and patient survival by Kaplan‐Meier analysis. We found that the overall survival time of the patients with high NDRG1 expression (n = 275) was longer than that of the patients with low NDRG1 expression (n = 370) (*P* = .001) (Figure 4J). Consistently, compared with patients with low mRNA level of NDRG1 (n = 260), patients with high mRNA level of NDRG1 (n = 270) also had longer overall survival time (*P* = .007) according to the analysis of TCGA data (Figure 4K).

**TABLE 3:** Clinicopathological features of the patients and correlation with NDRG1 protein expression in TMA data

	Low expression	High expression	*P*‐value	Total
Age‐year				
Median	57	58	.886^a^	58
Range	15‐82	27‐82		15‐82
Age‐year‐no. (%)				
≤55	162 (44)	110 (40)	.303^b^	272 (42)
>55	207 (56)	166 (60)		373 (58)
Gender‐no. (%)				
Male	261 (71)	187 (68)	.488^b^	448 (69)
Female	109 (29)	88 (32)		197 (31)
Fuhrman Grade‐no. (%)				
I + II	250 (68)	214 (78)	.006^b^	464 (72)
III + IV	119 (32)	62 (22)		181 (28)
TNM stage‐no. (%)				
I + II	305 (82)	244 (89)	.026^b^	549 (85)
III + IV	65 (18)	31 (11)		96 (15)
pT stage‐no. (%)				
T1 + 2	313 (85)	256 (93)	.001^b^	569 (88)
T3 + 4	57 (15)	19 (7)		76 (12)
pN stage‐no. (%)				
N0	355 (96)	266 (97)	.604^b^	621 (96)
N1	15 (4)	9 (3)		24 (4)
pM stage‐no. (%)				
M0	330 (89)	259 (94)	.026^b^	589 (91)
M1	40 (11)	16 (6)		56 (9)
Tumour burden‐cm				
Median	4.3	4	.08^a^	4
Range	0.8‐14	0.8‐13		0.8‐14
Tumour burden‐cm‐no. (%)				
≤4	179 (53)	190 (61)	.026^b^	369 (57)
>4	156 (47)	120 (39)		276 (43)

^a^ Mann‐Whitney U test.

^b^ chi‐square test; TNM stage: AJCC renal cancer in 2010.

**TABLE 4:** Clinicopathological features of the patients and correlation with NDRG1 mRNA expression in TCGA data

	Low expression	High expression	*P*‐value	Total
Age‐year				
Median	60	61	.642^a^	61
Range	32‐90	26‐88		26‐90
Age‐year‐no. (%)				
≤60	131 (50)	133 (49)	.796^b^	264 (50)
>60	129 (50)	137 (51)		266 (50)
Gender‐no. (%)				
Male	194 (75)	150 (56)	<.001^b^	344 (65)
Female	66 (25)	120 (44)		186 (35)
Fuhrman Grade‐no. (%)				
I + II	105 (42)	136 (51)	.038^b^	241 (46)
III + IV	148 (58)	133 (49)		281 (54)
TNM stage‐no. (%)				
I + II	151 (58)	171 (63)	.215^b^	322 (61)
III + IV	109 (42)	99 (37)		208 (39)
pT stage‐no. (%)				
T1 + 2	157 (60)	183 (68)	.076^b^	340 (64)
T3 + 4	103 (40)	87 (32)		190 (34)
pN stage‐no. (%)				
N0	250 (96)	263 (97)	.413^b^	513 (97)
N1	10 (4)	7 (3)		17 (3)
pM stage‐no. (%)				
M0	211 (81)	236 (88)	.046^b^	447 (85)
M1	48 (19)	33 (12)		81 (15)

^a^ Mann‐Whitney U test.

^b^ chi‐square test; TNM stage: AJCC renal cancer in 2010.

We next analysed the correlation between NDRG1 expression and metastasis based on the follow‐up information and TMA data set by linear‐by‐linear association. The incidence of metastasis in initially diagnosed patients was significantly lower in high NDRG1 expression patient than that in low NDRG1 expression ones (*P* = .021) (n = 645; Figure 4L). When excluding initially diagnosed ccRCC patients with metastasis, the patients with low NDRG1 expression were apt to have metastasis after radical nephrectomy (*P* = .015) (n = 589; Figure 4M).

### 3.5 NDRG1 suppressed the proliferation and metastasis of ccRCC tumour cells both in vitro and vivo

**FIGURE 5:**
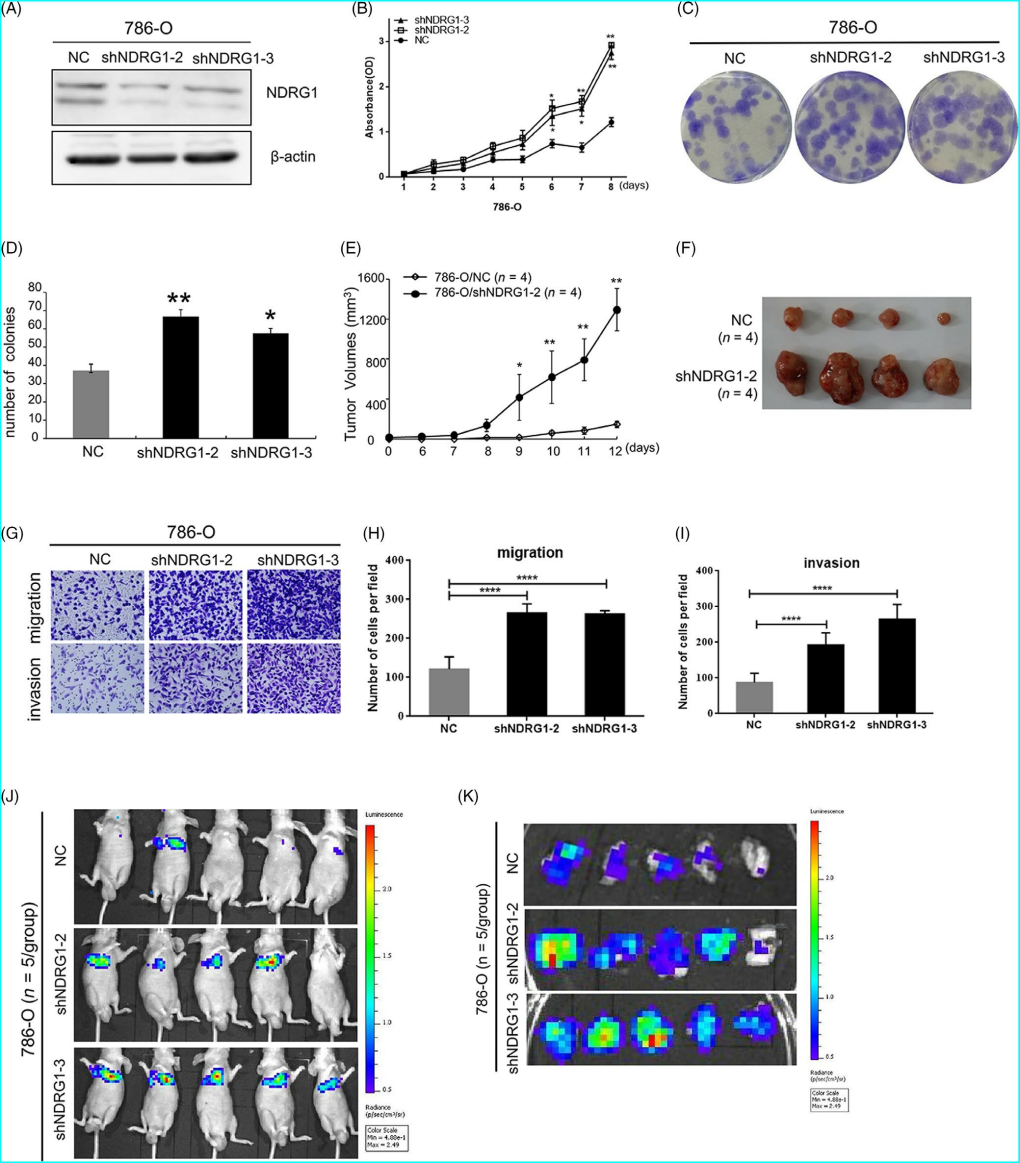
NDRG1 suppressed the proliferation and metastasis of ccRCC tumour cells. (A) Western blot for 786‐O control and NDRG1 shRNA cell lines, β‐actin as loading control. NC: 786‐O control cells; (B) NDRG1 inhibited cell proliferation as shown by CCK‐8 assays. (C and D) Colony formation assay indicated that NDRG1 knockdown significantly increased the cloning number of 786‐O cells compared with control group. (C) Representative image of the colony formation assay. (D) was quantification of (C). (E)Tumour growth curve and the tumour volumes were measured at different days after injection 786‐O cells stably transfected with control or shNDRG1. (F) Mice were sacrificed, and the subcutaneous tumours were removed (n = 4 for each group). (G) Migration and invasion assay were conducted to detect the migratory and invasive ability of 786‐O control cells and shNDRG1 cells. Each representative image is shown. (H and I) Quantitative results are, respectively, illustrated for Transwell assay. (J) Representative images of mice in each group at 28 d after tail vein injections of 786O cells (control or shNDRG1‐2/3 infected, n = 5/group). (K) Representative images of pulmonary metastasis in each group at 28 days after tail vein injections. All data represented the mean ± SD from three independent experiments, *P < .05, **P < .01, ****P < .0001 for unpaired t test

To investigate the biological role of NDRG1 in tumour formation, we introduced two independent NDRG1 shRNAs into 786‐O cells with relatively high expression of NDRG1 (Figure 5A). The growth of the 786‐O cells transfected with NDRG1‐shRNAs was increased compared with the control (Figure 5B). Similarly, impaired NDRG1 expression increased the colony formation capacities of ccRCC cells (Figure 5C and D). In addition, we introduced NDRG1 into Caki‐1 cells that have low constitutive expression of NDRG1 (Figure S1A). Then, we found that the growth of Caki‐1 cells transfected with NDRG1 was suppressed relative to that of the control cells by CCK8 assays (Figure S1B). And the number of Caki‐1/NDRG1 cells was significantly less than that of Caki‐1/EV cells through colony formation assay, which confirmed the ability of NDRG1 to inhibit the colony formation capacities of Caki‐1 cells (Figure S1C and D). To further examine the impact of NDRG1 in vivo, we implanted nude mice with shNDRG1 transfected 786‐O cells and control cells (n = 4 for each group). The dynamic quantitative tumour volume of the shNDRG1 group was consistently bigger than that of the control group (Figure 5E). Compared with the shNDRG1 group, the tumours derived from the control 786‐O cells were obviously smaller after two weeks (Figure 5F). Our work has highlighted the key role of the NDRG1 in suppressing the proliferation of ccRCC tumour cells.

As shown in Figure 5G, H and I, NDRG1 significantly decreased the migration and invasion capabilities of 786‐O cells by Transwell assay. The inhibitory effect of NDRG1 on metastasis in vitro has also been confirmed in Caki‐1 cells (Figure S1e, f and g). Next, we performed the tail vein metastasis assay to test the inhibitory effect on metastasis in *vivo*. Luciferase‐labelled control and 786‐OshNDRG1 cells were injected into the tail veins of nude mice (n = 5 for each group). Compared with control group, luciferase signals in the mice from 786‐OshNDRG1‐2/3 groups were significantly higher at 28 days after injection (Figure 5J), which was consistent with more foci in the lungs of the mice in 786‐OshNDRG1‐2/3 groups (Figure 5K). These results suggested that NDRG1 suppressed metastasis of ccRCC cells. It may be that the growth of 786‐O/shNDRG1‐2/3 cells was faster, which resulted in stronger metastasis in the lungs of mice.

### 3.6 High HIF‐1/2α predicted a poor prognosis in ccRCC after adjustment of the expression of NDRG1

**FIGURE 6:**
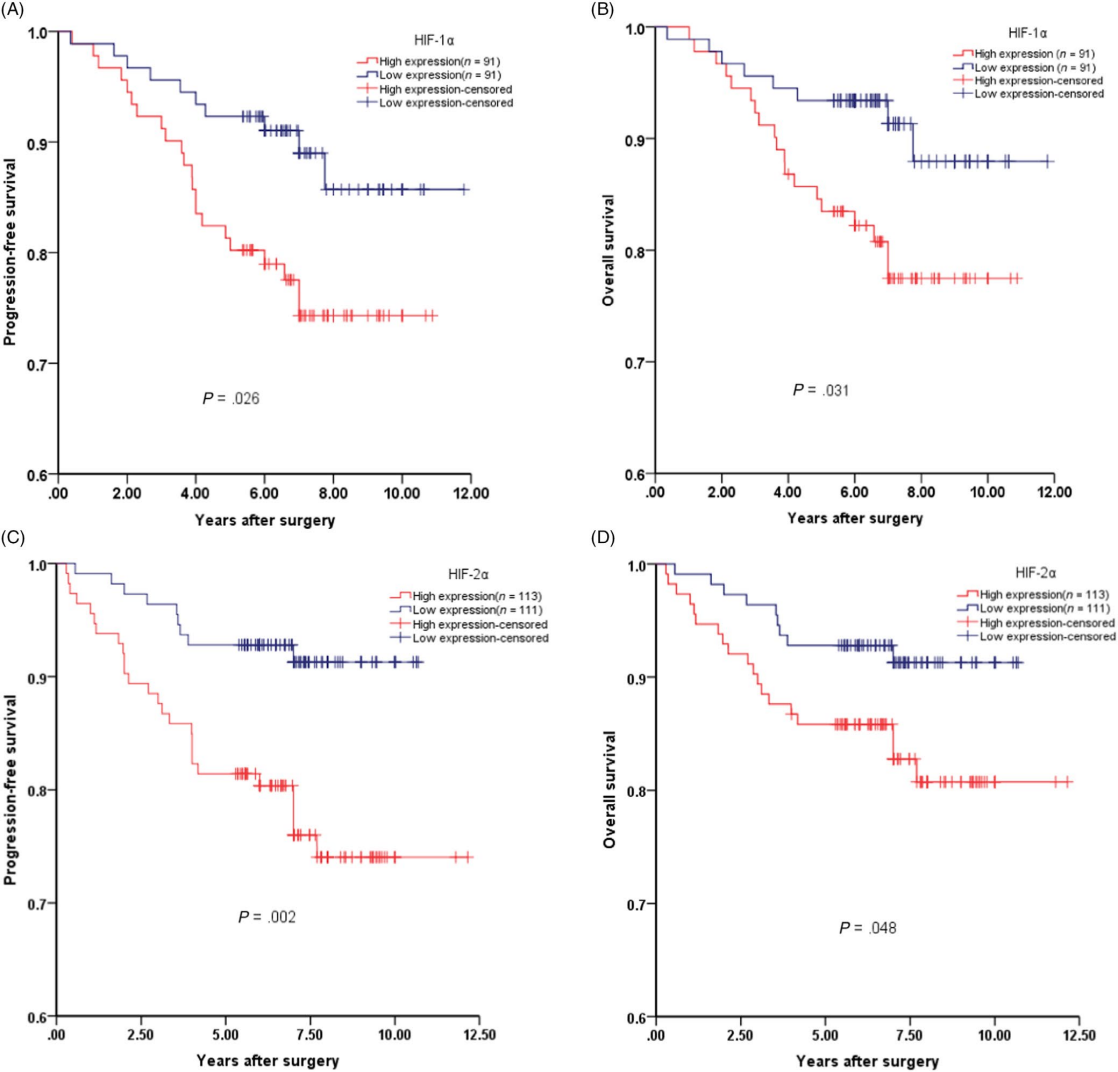
High HIF‐1/2α predicted a poor prognosis in ccRCC after adjustment of the NDRG1 expression. (A) Progression‐free survival and (B) overall survival among ccRCC patients with low or high protein levels of HIF‐1α by Kaplan‐Meier analysis. (C) Progression‐free survival and (D) overall survival among ccRCC patients with low or high protein levels of HIF‐2α by Kaplan‐Meier analysis. The log rank *P*‐value is reported

As shown in Figure 1, there was no significant difference in PFS and OS between the high‐ and low‐expression groups of HIF‐1/2α. However, the expression level of NDRG1 in ccRCC patients with high levels of HIF‐1/2α protein was significantly higher than that in ccRCC patients with low levels of HIF‐1/2α protein (Figure S2a and b). In addition, the higher NDRG1 was found to suppress the development of ccRCC. Next, we adjusted ccRCC patients in high‐HIF‐1/2α level group and low‐HIF‐1/2α level group with the NDRG1 expression level so that there was no significant difference in the expression of NDRG1 between the two ccRCC groups (Figure S2c and d). And then, we found that the PFS and OS time of the patients with high‐HIF‐1α protein level (n = 91) were significantly shorter than that of the patients with low‐HIF‐1α protein level (n = 91) (PFS, *P* = .026; OS, *P* = .031) (Figure 6A and B). Consistently, compared with patients with low‐HIF‐2α protein level (n = 111), patients with high‐HIF‐2α protein level (n = 113) also had significantly shorter PFS and OS time (PFS, *P* = .002; OS, *P* = .048) (Figure 6C and D). These results suggested that the up‐regulation of NDRG1 by HIF counteracts the cancer‐promoting effect of HIF in ccRCC patients.

### 3.7 NDRG1 suppressed the expression of oncogenes in ccRCC cells

**FIGURE 7:**
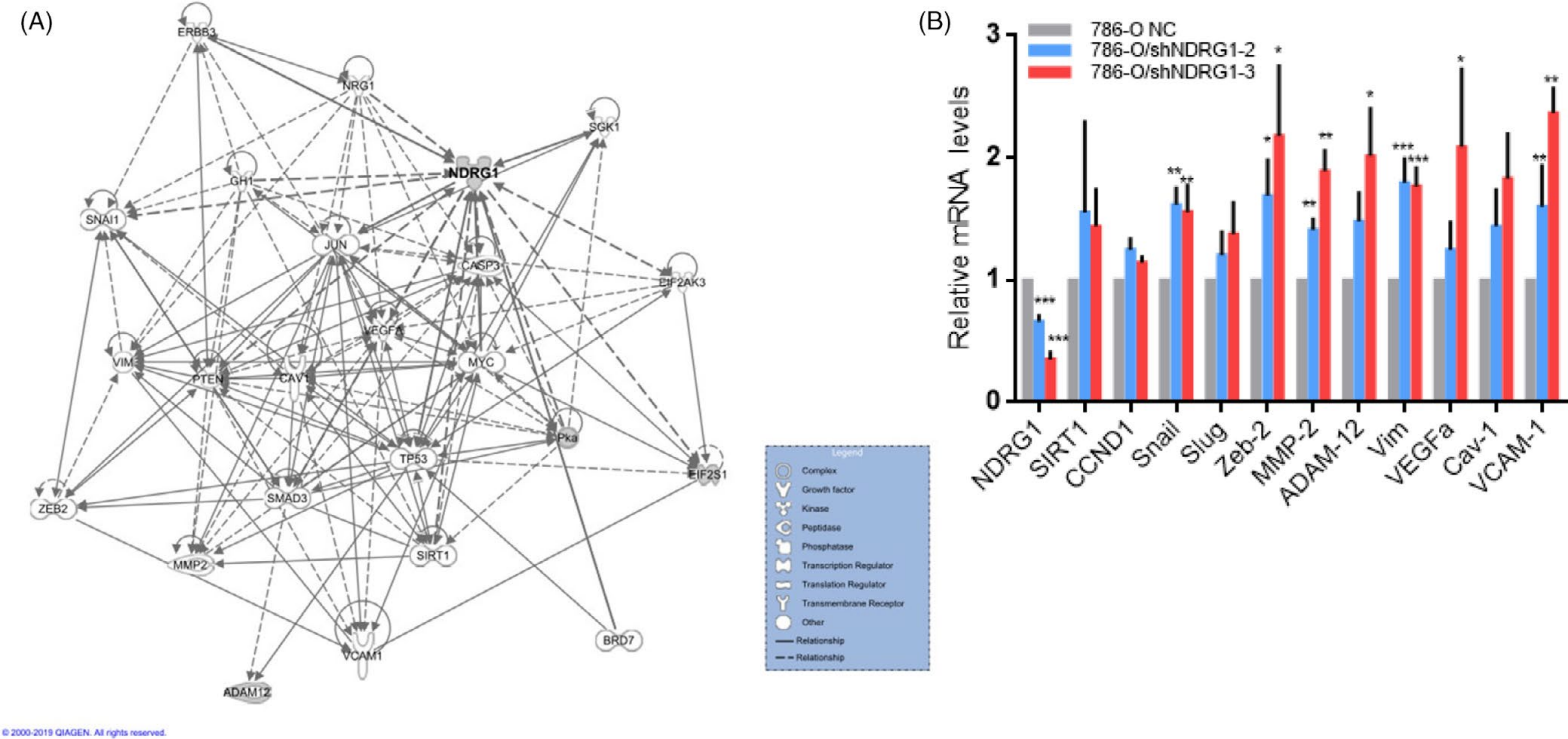
NDRG1 suppressed the expression of oncogenes in 786‐O cells. (A) The interaction map showed gene‐gene interaction network generated by using IPA for NDRG1 and genes participating in the tumour proliferation and metastasis. (B) Effects of NDRG1 knockdown (shNDRG1‐2 and shNDRG1‐3) on gene expression as measured by qRT‐PCR analysis in 786‐O cells. Expression was normalized to the cells with the shRNA control vector. Data were shown as the mean ± SEM of three independent experiments. (*P < .05, **P < .01, ***P < .005 for unpaired *t* test)

To screen the clues of NDRG1 suppressing cancer, we performed an interaction analysis of NDRG1 with the cancer proliferation‐ and metastasis‐associated genes from the IPA database (Figure 7A). The pathways and gene‐regulatory network constructions demonstrated that NDRG1 was a node of the network and a set of genes tightly interacted with NDRG1. We further confirmed the expression of oncogenes by qRT‐PCR. In the Figure 7B, the expression of cyclin D1 (CCND1), sirtuin 1 (SIRT1), Snail, Slug, Zeb‐2, MMP‐2, ADAM‐12, VEGFa, Vim, Cav‐1 and VCAM‐1 was increased when NDRG1 was knocked down in 786‐O cells. Next, we ectopically expressed NDRG1 in Caki‐1 cells to verify the effect of NDRG1 on the expression of these oncogenes. As shown in the Figure S3, the expression of Snail, Slug, MMP‐2, ADAM‐12, Vim, VEGFa and VCAM‐1 was suppressed when NDRG1 was overexpressed. This suggests that NDRG1 inhibits tumour proliferation and metastasis by suppressing the expression of genes involved in carcinogenesis.

## 4 DISCUSSION

As documented in the past decades, HIF‐1/2α is thought to be deeply involved in the carcinogenesis and progression of tumours and the expression of HIF‐1/2α has been supposed to be tight with the prognosis in various cancers.^6^, ^7^ In our study, the bioinformatic analysis of putative genes regulated by HIF‐1/2α in 786‐O cells demonstrated that the HIF‐1/2α should be associated with poor prognosis of ccRCC.

However, the implication of HIF‐1/2α in the prognosis of ccRCC is controversial and largely unclear. Piotr M et al have shown that HIF‐1/2α expression is associated with an inferior survival.^19^, ^20^ In contrast, it have been reported that HIF‐1α expression in ccRCC was associated with the good prognosis, and HIF‐1α acts as a tumour suppressor in ccRCC.^21^ We collected large set of tumour tissue specimens and follow‐up information of 331 ccRCC patients. Surprisingly, we found no correlation between HIF‐1/2α protein expression and outcome of patients. The inconsistence between these other studies and ours may be caused by the relative smaller sample size in these other studies. Our work may provide relatively credible evidence for the role of HIF‐1/2α in ccRCC prognosis because of the large set of tumour tissue specimens.

Our findings did not demonstrate the cancer‐promoting effect of HIF. It suggested that some genes downstream of HIF‐1/2α may act as a tumour suppressor and counteract the cancer‐promoting effect of HIF‐1/2α. We identified the common genes regulated both by VHL and hypoxia in RCC4 and 786‐O cell lines. The common genes that were regulated by both VHL and hypoxia may be potential HIF‐regulated genes. Finally, we identified NDRG1 as a candidate downstream of HIF to suppress tumour progression.

NDRG1 is down stream of n‐myc/c‐myc, and the later could suppress its expression. NDRG1 can be up‐regulated at protein and mRNA levels by hypoxia, cellular iron depletion and DNA damage through HIF‐1α‐dependent and ‐independent mechanisms.^22^, ^23^ However, its role is controversial in various tumours. In colon, prostate, breast and pancreatic cancer, NDRG1 suppresses tumour proliferation and metastasis.^24^, ^25^, ^26^, ^27^, ^28^ In contrast, NDRG1 stimulates carcinogenesis in tumours of the liver, bladder, oesophagus and cervix.^29^, ^30^, ^31^, ^32^ However, its role in ccRCC is largely unknown.

We constructed the interaction comprehensive networks between NDRG1 and VHL‐regulated genes and annotated these with biological function. NDRG1 plays a central role in regulatory networks and interacted with JUN, STAT3, CXCL5, XRCC6 and IL4, which involve in carcinogenesis and metastasis.^33^, ^34^, ^35^, ^36^, ^37^ In addition, NDRG1 was shown to be down‐regulated by VHL and involved in carcinoma, apoptosis, cell death and migration of tumour cell lines.

We further verified that NDRG1 was regulated by both HIF‐1 and −2α in ccRCC cell lines, and the expression level of NDRG1 protein was also positively correlated with HIF‐1/2α in ccRCC tumour tissues. NDRG1 protein was significantly increased in ccRCC cancer tissues compared to paired normal renal tissues. And NDRG1 protein in the cytoplasm suggested favourable prognosis from our large set of clinical data. CCK8 proliferation assay in vitro and subcutaneous tumour formation in mice confirmed the role of NDRG1 in inhibiting tumour proliferation. Transwell assay in vitro and mouse tail vein assay suggested that NDRG1 suppressed tumour metastasis. Notably, when the expression level of NDRG1 was adjusted, the high‐HIF group patients had a much worse survival than the low‐HIF group ones (Figure 6). Our study provided direct evidences that NDRG1 might be a tumour suppressor in ccRCC. Intriguingly, Hosoya et al's study suggested the high nuclear NDRG1 protein predicted a favourable prognosis.

In addition, it has been reported that the downstream genes of HIF play an anti‐tumour role in ccRCC.^38^, ^39^ For example, Zhang et al reported that GLUT1 and IGFBP3, which were induced by HIF, functioned as tumour suppressive genes in ccRCC.^38^ IRF9 and STAT2, as components of interferon stimulated gene factor 3 (ISGF3), are up‐regulated by HIF‐2α in 786‐O cells. And ISGF3 played an anti‐cancer in a ccRCC xenograft model.^39^

And we obtained the molecular mechanisms of NDRG1 by comparing 786‐O‐shNDRG1‐2/3 cell with 786‐O control cells and comparing Caki‐1/EV with Caki‐1/NDRG1. We found that the expression of CCND1, SIRT1, Snail, Slug, Zeb‐2, MMP‐2, ADAM‐12, VEGFα, Vim, Cav‐1 and VCAM‐1 was suppressed by NDRG1. CCND1 is a key regulator of G1/S transition and cell proliferation. SIRT1 regulates ageing and resistance to oxidative and DNA damage stress by inhibiting cellular apoptosis or senescence. Consistent with their role, in cancer cells, they act as oncogenes by promoting tumorigenesis.^40^, ^41^ It is best known that about 90% of cancer‐related deaths are caused by metastatic disease rather than primary tumours.^42^ The characteristic that malignant tumour cells have the ability of metastasis is mainly achieved through epithelial‐mesenchymal transition (EMT). EMT is a series of cell‐biological programmes coordinated by master EMT‐inducing transcription factors (EMT‐TFs), especially Snail, Slug and Zeb2.^43^ In addition, VEGFα, MMP2, ADAM12 and Vim, which are produced by cancer cells, have been reported to disrupt vascular integrity. They have previously been beneficial during primary tumour invasion and have proven useful at the invasion‐metastasis cascade.^44^ VCAM‐1‐expressing carcinoma cells are able to obtain the ability to metastatic colonization by activating AKT signalling.^45^ The modulated profiling of these proliferation and metastasis‐related genes in NDRG1‐silencing cells indicated the potential molecular events downstream of NDRG1. It has been reported that NDRG1 and the mitogen‐inducible gene 6 (MIG6) form a complex through direct interaction in the cytoplasm, which facilitates lysosomal process of epidermal growth factor receptor (EGFR) and down‐regulates the EGFR expression.^46^, ^47^ Thus, the oncogenic PI3K/AKT,^48^ WNT^49^ and NF‐κBand TGF‐β^28^ signalling pathways could be down‐regulated by NDRG1 via EGFR. This suggests that NDRG1 may be involved in the expression of theses oncogene via indirect ways.

In this work, we used proteomics to screen for genes regulated by hypoxia and VHL, which did not fully represent the downstream genes of HIF‐1/2α. So, there may be other tumour suppressor genes downstream of HIF‐1/2α that we missed. In addition, both HIF‐1α and HIF‐2α can up‐regulate NDRG1 expression; thus, further research is necessary to determine the different ability of HIF‐1α and HIF‐2α in ccRCC on regulating NDRG1.

In conclusion, we demonstrated HIF downstream gene of NDRG1 counteracts the cancer‐promoting effect of HIF. And we revealed the expression pattern, biological function and potential regulatory mechanism of NDRG1 in ccRCC. These results provide evidence that NDRG1 may be a potential prognostic biomarker as well as a therapeutic target in ccRCC.

## CONFLICT OF INTEREST

The authors declare that they have no competing interests.

## AUTHORS’ CONTRIBUTIONS

LSW, WZ and ZYZ conceived and designed the study. ZYZ, HLC, YQM, JZ, YHC and ZML performed the experiments. CYK, HLC, SLZ, BH and WX analysed and interpreted the data. ZYZ and LSW wrote the draft of the manuscript and all authors revised the manuscript.

## ETHICAL APPROVAL

All human sample‐related studies have been approved by the ethics committee of Shanghai Renji Hospital. Animal care and experiments were carried out in strict accordance with the Animal Care and Use Guide and approved by the ethics committee of Fudan University.

## Supporting information

Data S1

Data S2

Data S3

Data S4

Data S5

Data S6

Data S7

Data S8

Data S9

Fig S1

Fig S2

Fig S3

## Data Availability

The data generated or analysed during this study are included in this article or if absent are available from the corresponding author upon reasonable request.
