# Supplementary figures and images for: The up‐regulation of NDRG1 by HIF counteracts the cancer‐promoting effect of HIF in VHL‐deficient clear cell renal cell carcinoma
Source: Cell Prolif. 2020 Jun 14;53(7):e12853. doi: 10.1111/cpr.12853 (PMC7377940; doi:10.1111/cpr.12853)

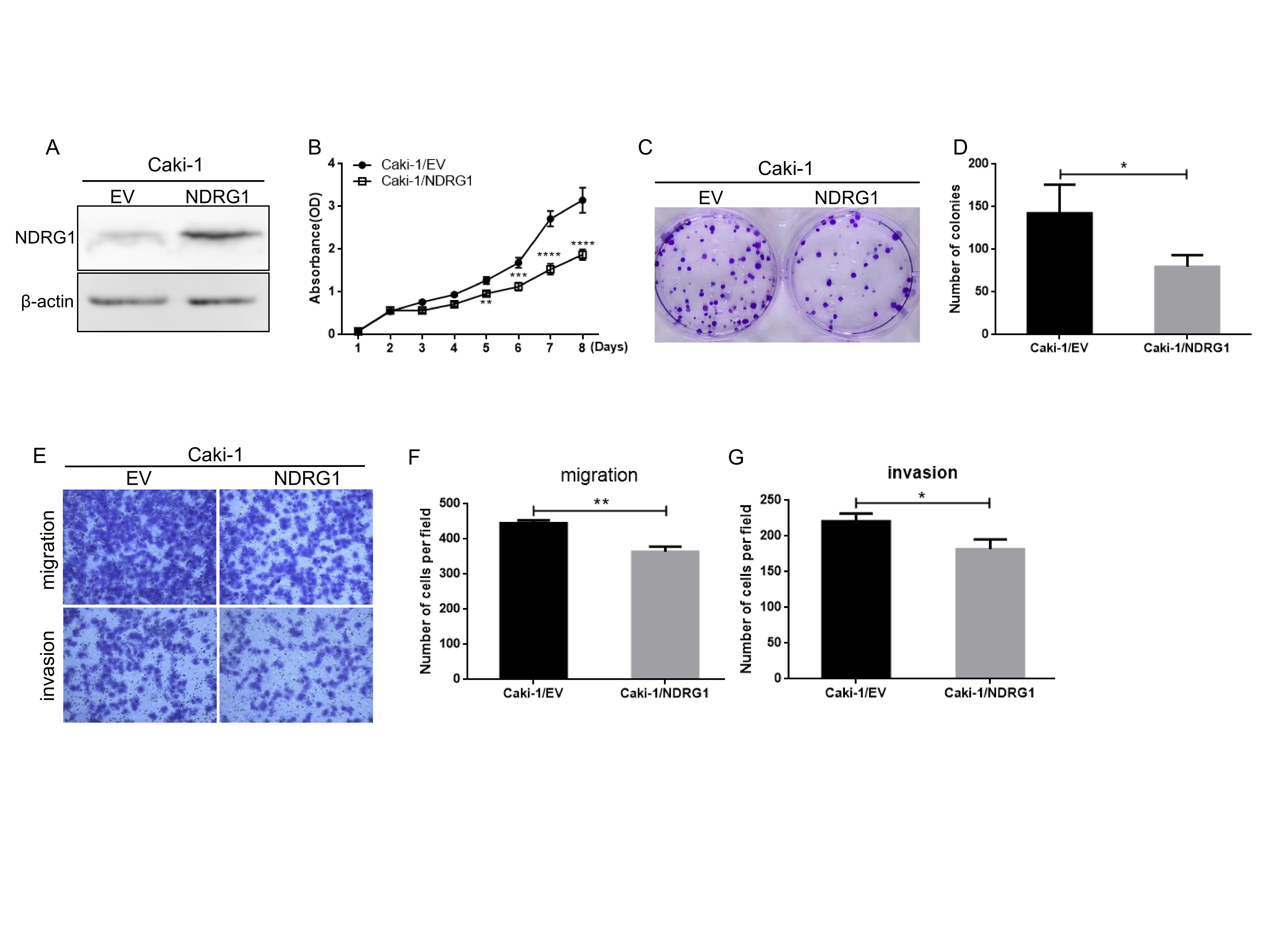

Supplement: Supplementary file 10 — Fig S1 [file CPR-53-e12853-s010.tif]

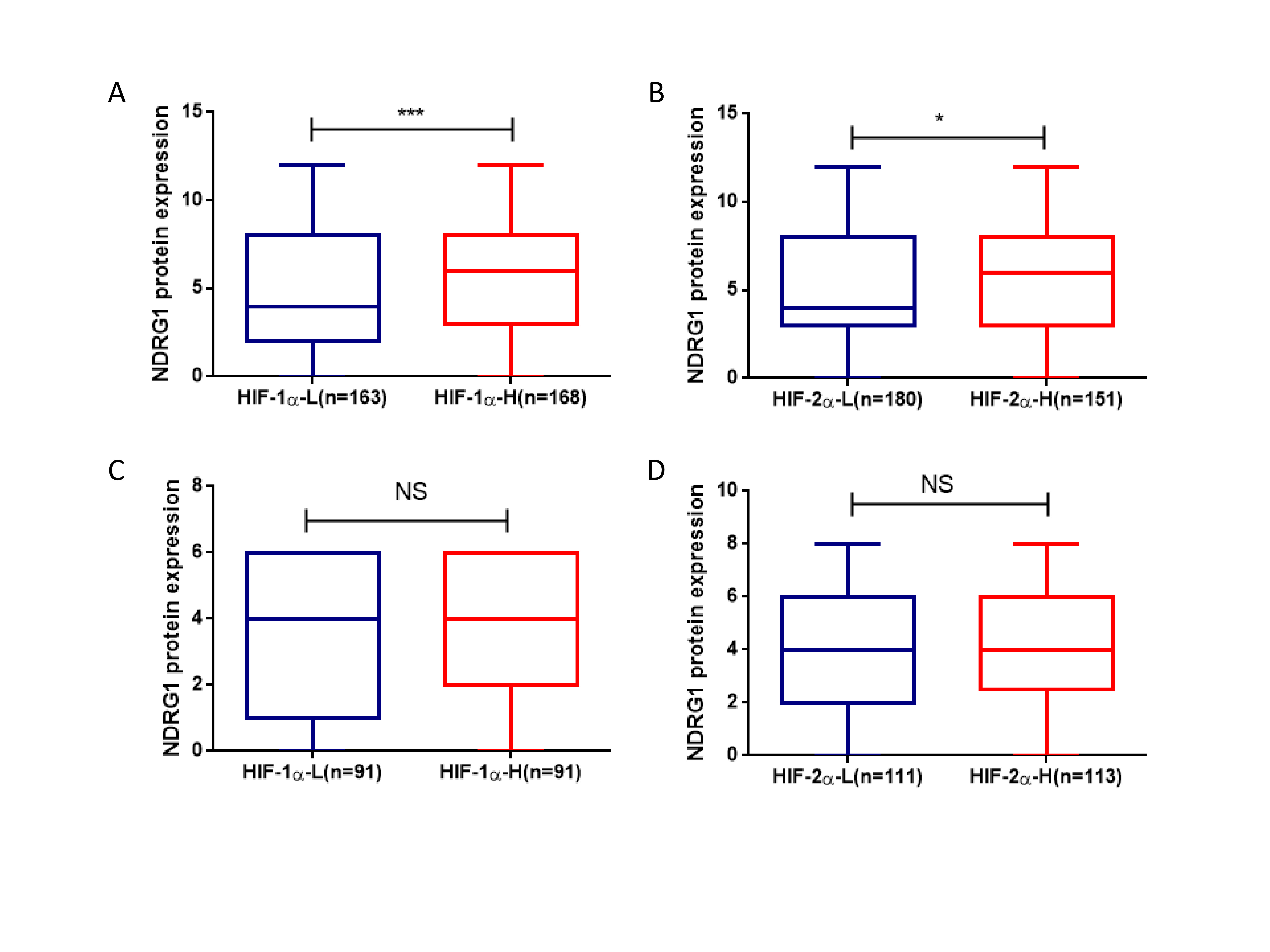

Supplement: Supplementary file 11 — Fig S2 [file CPR-53-e12853-s011.tif]

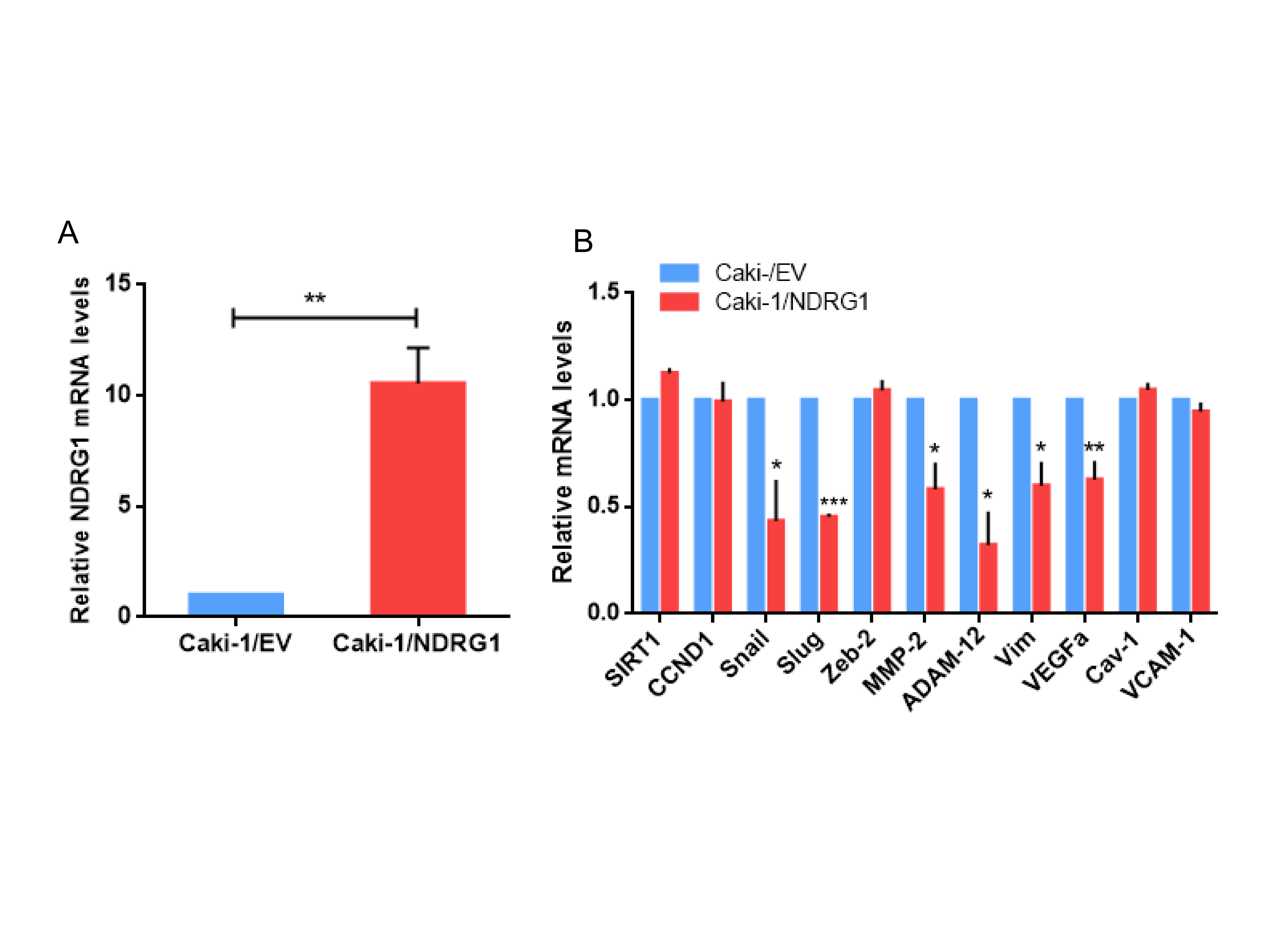

Supplement: Supplementary file 12 — Fig S3 [file CPR-53-e12853-s012.tif]
